# Near Point-of-Care HIV Viral Load: Targeted Testing at Large Facilities

**DOI:** 10.1097/QAI.0000000000002555

**Published:** 2020-10-21

**Authors:** Prakash Ganesh, Tom Heller, Boniface Chione, Joe Gumulira, Salem Gugsa, Shaukat Khan, Seth McGovern, Angellina Nhlema, Lyse Nkhoma, Jilian A. Sacks, Clement Trapence, Hannock Tweya, Peter Ehrenkranz, Sam Phiri

**Affiliations:** a Lighthouse Trust, Lilongwe, Malawi;; b International Training and Education Center for Health, University of Washington, Seattle, WA;; c Clinton Health Access Initiative, Boston, MA;; d Global Health, Bill and Melinda Gates Foundation, Seattle;; e Department of Medicine, University of North Carolina School of Medicine, Chapel Hill, NC;and; f Department of Public Health, College of Medicine, School of Public Health and Family Medicine, University of Malawi, Zomba, Malawi.

**Keywords:** HIV, point of care, targeted viral load, cost analysis, GeneXpert

## Abstract

Point-of-care (POC) technologies in resource-limited settings can circumvent challenges of centralized laboratory testing, improving clinical management. However, higher device costs and uncertain indications for use have inhibited scaling up POC modalities. To address this gap, we investigated the feasibility and cost of targeted near-POC viral load (VL) testing in 2 large HIV clinics in Lilongwe, Malawi.

**Methods:**

VL testing using GeneXpert was targeted for patients suspected of treatment failure or returning to care after a previously elevated VL (>1000 copies/mL). Descriptive analysis of retrospective clinical and cost data is presented.

**Results:**

Two thousand eight hundred thirteen near-POC VL tests were conducted. One thousand five hundred eleven (54%) tests were for patients for whom results and reason for the test were documented: 57% (794/1389) of tests were to confirm a previously high VL, and 33% (462/1389) were due to clinical indications. Sixty-one percent (926/1511) of patients had a high VL, of whom 78% (719/926) had a recorded clinical action: 77% (557/719) switched to second line antiretroviral therapy, and 15% (194/719) were referred for intensive adherence counseling. Eighty-two percent (567/687) of patients received a clinical action on the same day as testing. The “all-in” cost was $33.71 for a valid POC VL test, compared with an international benchmark for a centralized VL test of $28.62.

**Conclusion:**

Targeted, near-POC VL testing was feasible and consistently enabled prompt clinical action. The difference between the “all-in” cost of near-POC VL and centralized testing of $5.09 could be further reduced in an optimized national program by combining targeted near-POC testing and centralized testing.

## INTRODUCTION

In 2013, the World Health Organization (WHO) recommended universal access to antiretroviral therapy (ART) and routine viral load (VL) testing to monitor treatment efficacy.^1^ This global guidance aligns with UNAIDS 90-90-90 targets to be achieved by 2020: 90% of people living with HIV (PLHIV) know their status, 90% of people who know their status are accessing treatment, and 90% of people on treatment are virally suppressed.^2^ In 2017, it was estimated that there were 36.9 million PLHIV with 21.7 million accessing ART.^3^ Although much global effort has been made to increase the uptake and coverage of ART, there is a gap in viral suppression targets, especially in Eastern and Southern Africa, where in 2017, only 52% of all PLHIV were virally suppressed.^4^ Of the 70% of PLHIV in sub-Saharan Africa, more than 6 million are on ART but many lack access to VL testing.^5,6^

The WHO guidelines recommend VL testing at 6 and 12 months after ART initiation, and then annually thereafter.^1^ However, laboratory capacity to conduct VL monitoring varies across sub-Saharan Africa. Ninety-one percent of PLHIV on ART in South Africa had at least one VL performed in 2015,^7^ whereas only 53% of PLHIV on ART in Malawi met their monitoring milestone by the end of 2016.^8^ Enabling routine access to VL testing to ensure viral suppression is necessary to reduce the HIV burden and associated morbidity and mortality,^9,10^ but testing coverage is only impactful if results are used appropriately to differentiate patients to the model of care that meets their condition.^11^ In many countries, PLHIV on ART for at least 1 year with a suppressed VL (<1000 copies/mL) are considered stable and eligible for a less intense model of care, which could include decreased dispensing frequency or participation in a community-based adherence club. Conversely, PLHIV with an unsuppressed VL (>1000 copies/mL) require more intensified care that includes adherence counseling and subsequent regimen change if the VL does not resuppress with time.^1,12^ Despite these recommendations, many stable patients unnecessarily remain in more intense models of care,^13^ and the rates of ART regimen switch to second line do not correspond well to the estimated rates of unsuppressed VL, despite the known risk of poor outcomes.^14,15^

One proven approach to increase access to testing services and rates of clinical action is point-of-care (POC) technologies—tests that have rapid turnaround times (TAT) that enable result availability and clinical action in the same visit.^16^ These tests can be operated outside of conventional laboratory settings by laboratory or nonlaboratory personnel and closer to locations where patients are receiving care, either in the facility or the community. POC tests range from device-free rapid HIV or pregnancy tests to device-based near-POC technologies, such as the GeneXpert (Cepheid, Sunnyvale, CA). By enabling testing on-site, near-POC technologies can remove the need for specimen transport to centralized laboratories that lead to long TAT between sample collection and result availability—nearly 7 weeks in some settings.^7^ However, decentralized testing brings additional challenges, including the need for decentralized quality control, equipment maintenance, supply chain, and waste management.^16,17^ In addition, there is a perception of increased expense because the cost for POC proprietary commodities and devices may be higher than those for centralized laboratory machines while the throughput may be substantially lower.^18^

Despite these challenges, the evidence that near-POC testing improves the management of PLHIV continues to grow. Near-POC CD4 testing has been successfully rolled out in resource-limited settings for the past decade, resulting in increased ART initiations and retention in care.^19^ Similarly, POC testing for early infant diagnosis (EID) has been shown to increase rates of ART initiation for HIV-positive infants across several countries in sub-Saharan Africa.^20–22^ Evidence is emerging on how near-POC testing could benefit VL testing programs. A recently completed study in South Africa replaced centralized testing with near-POC VL within a package of services for adult clients newly initiating efavirenz-based ART. The combination intervention increased the proportion of patients achieving viral suppression, retained in care, and referred into community-based care.^23^ In addition, a recent project in Malawi demonstrated that routine POC VL testing decreased overall TAT, time to intensified adherence counseling, and a shorter time to switch to second line for patients with a VL >1000 copies.^24^

In the resource-limited setting of Malawi, we hypothesized that an optimal testing program for nonpregnant or breastfeeding patients would combine (1) high-volume centralized testing for most patients requiring routine VL assessments with (2) targeted near-POC VL testing on low throughput machines for patients whose management requires the same day result, specifically patients suspected of treatment failure or returning to care after a previously elevated VL (>1000 copies/mL). The strategy was predicated on the evidence that most patients on ART are effectively managed through centralized VL testing, despite extended TAT for results. National data show that 91% of PLHIV on ART are virally suppressed^25^ and nearly 70% are receiving 3 monthly refills^13^ so typically receive their VL result on their next visit or are called by phone if the test result is unsuppressed.

To improve the management of those who were at high risk for being unsuppressed, we provided targeted near-POC VL testing to eligible patients at 2 high-volume facilities in Lilongwe and assessed the process and cost implications in a programmatic setting.

## METHODS

### Setting

The prevalence of HIV infection among adults aged 15–64 years in Malawi is estimated at 10.6%, and 67.6% of all PLHIV are virally suppressed. ^25^ During the time of this project, the country guidelines recommended VL monitoring at 6 months and at 2 years after ART initiation and every 24 months thereafter, which differs from the WHO recommendations.^12^ The decision to reduce the frequency of treatment monitoring in Malawi was driven by a desire to maximize coverage while minimizing costs.^26^ This guidance was changed to annual VL monitoring in 2019. In addition, the national ART guidelines recommend regimens based on 2 nucleoside reverse transcriptase inhibitors (NRTIs), combined with a non-NRTI, mainly efavirenz as first-line ART. As second-line ART, a combination of 2 NRTIs with ritonavir-boosted protease inhibitors are recommended. Confirmed treatment failure occurs after a targeted or repeat VL is >1000 copies/mL, with good adherence in the previous 3 months. After a switch to second-line or third-line ART, patients reset their clock for routine VL monitoring.^12^

The Lighthouse (LH) Trust is a public trust and a WHO-recognized Center of Excellence for integrated HIV prevention, treatment, care, and support partly funded through the Malawi Ministry of Health but receives significant supplemental funding from the President’s Emergency Plan for AIDS Relief through the Centers for Disease Control and Prevention. LH operates 2 large urban patient-centered HIV clinics in Lilongwe: one at the Kamuzu Central Hospital (LH-KCH) with a current cohort of 10,729 patients, and the second called the Martin Preuss Center (LH-MPC) with 21,701 patients.

### Intervention

#### Implementation of Near-POC VL Testing Using GeneXpert

From January to December 2017, the GeneXpert HIV-1 VL assay was available at the 2 LH facilities for nonpregnant adult and adolescent clients who met the following criteria: clinically suspected of ART failure or advanced HIV disease, such as those with WHO stage 3 and 4 diseases or those with low CD4 counts; in need of follow-up because of previous high VL; and switched to second-line ART 6 months previously, regardless of clinical status. Clinicians were trained on testing guidelines for VL monitoring and failure management in January 2017.

One GeneXpert platform with 4 modules was placed in each clinic and was primarily operated by nurses. A laboratory technologist also operated the GeneXpert platform at LH-KCH and assisted with supply chain management and technical support for both sites. At LH-KCH, most tests were for VL (94%; 1364/1445), and at LH-MPC, the majority of tests were for VL (58%; 1449/2489).

The Xpert HIV-1 VL test, which is run on the GeneXpert platform (Cepheid, Sunnyvale, CA)^27^ is approved by the European Union and WHO Prequalification Program. The GeneXpert technology enables the automated quantitation of HIV VL, requiring 1 mL of plasma in 104^27^ minutes. The assay has been validated in lower resourced clinical settings and detects virological failure (>1000 copies per mL) with 94% sensitivity and 99% specificity.^28,29^

### Data Collection and Analysis

Near-POC VL testing and results data were extracted from the POC laboratory registers and POC VL request forms. LH clinics serve both patients within their primary cohort on-site and referral patients. VL results for referred patients are returned directly back to the patient without maintaining documentation at LH facilities; therefore, the data analysis was restricted to the primary cohort. Demographic and clinical data were abstracted from the electronic medical record system. Process outcomes from the targeted near-POC testing included accuracy of pretest risk assessment of treatment failure, time to clinical action, and costs to implement the targeted near-POC VL testing. Data were analyzed using Stata 14.2.

As the targeted POC VL testing was implemented concurrently with an aggressive process to ensure all LH patients received at least one VL test, an excess number of routine centralized VL tests among LH patients occurred during the study period. Comparisons of the targeted approach with a routine testing approach within the same cohort were therefore not possible.

Missing values were evaluated using Little's missing completely at random test and did not show any systematic patterns in missing data (*P* > 0.5). There were also no associations between categorical variables and missingness.

#### Costing Model

Retrospective data on the costs incurred during the study period to provide targeted near-POC VL testing services in the 2 clinics were collected, including the commodity cost of the tests and equipment as well as human resources, waste management, and facility overhead. These data were entered into the Health Economics and Epidemiology Research Office's Testing Technology Cost Model to analyze the “all-in” cost per test (Health Economics and Epidemiology Research Office. Testing Technology Cost Model. Johannesburg: HE2RO 2017). Where exact figures were not available, estimates from similar contexts were used. The study reported costs in the United States Dollar.

#### Costing Inputs and Assumptions

The following equipment was purchased for the purpose of this study: Cepheid GeneXpert IV device, service contract, centrifuge, and uninterrupted power supply. The total cost to set up a testing site for laboratory equipment was $22,316, with the majority $17,000 comprised the POC GeneXpert device. During the study, testing activities were conducted by 3 cadres of health workers who worked full time, senior nurses, junior nurses, and laboratory technicians. The model was populated with corresponding salary information, inclusive of benefits, for each cadre of health worker in a top-down costing approach. The materials required to run each test were GeneXpert HIV VL test cartridges; gloves, syringes, and EDTA tubes for specimen collection; and paper and toner for printing results. The prices for gloves, syringes, and EDTA tubes were reported from invoices from the study. The GeneXpert HIV VL cartridge was benchmarked to manufacturer's ex-works pricing. Toner and paper were assumed to be purchased at typical benchmark prices. There were additional costs incurred for freight, clearing and duty to import the test cartridges, consumables, and equipment; there was one initial training and one refresher training (not included in national scale-up scenario) conducted during the study period; waste management costs were incurred for the disposal of GeneXpert HIV VL, which contains guanidinium thiocyanate, a toxic substance. It was not possible to collect study-specific overhead data, so a fixed percentage of 15% was used across all other components of the all-in cost per test. There were assumed to be 20 working days in a month after accounting for weekends and holidays.

### Ethical Approvals

Targeted POC VL testing was provided for as routine care and followed the national guideline protocols; therefore, no informed consent was required. The protocol was approved by the Human Research Ethics Committee of the National Health Science Research Committee of Malawi.

## RESULTS

### Near-POC VL Testing

During the study period, a total of 2813 VL tests were run on the GeneXperts at the 2 study sites, with an error rate of 4% (59/1364) at LH-KCH and 6% (87/1449) at LH-MPC.

Of the 2813 POC VL tests conducted across both LH-KCH (n = 1364) and LH-MPC (n = 1449), 54% (1511) tests were for patients who received their primary HIV care at LH and thus had documented results, with the remainder testing volume for referred patients. Fifty-seven percent (n = 794/1389) of LH primary patients had a previous high VL, 33% (462/1389) patients had a clinical indication of advanced HIV disease, 3.6% (n = 50/1389) patients were being monitored after switching to second line, 6% (n = 83/1389) patients for other reasons, and 8% (n = 122/1511) patients were missing a reason for testing on their sample referral form and assumed to be missing completely at random (*P* > 0.5) (Table 1). Of the patients with a clinical indication, the most common signs and symptoms were loss of weight (42%; 193/462), followed by new WHO stage 3 or 4 disease (36%; 165/462), night sweats (21%; 96/462), and chronic cough (17%; 78/462) (Table 1). Sixty-one percent (n = 926/1511) of the patients with an available result had an elevated VL ≥1000 copies/mL (Table 1).

**TABLE 1.** Characteristics of POC VL Testing*

	N (%) (N = 1511)
Demographics	
Sex, female,	812 (61)
Age [mean (IQR)]	35 (27–44)
Reason for testing (n = 1511–122 missing = 1389)	
Clinical indication (multiple choices possible)	462 (33)
Loss of weight	193 (42)
Night sweats	96 (21)
Chronic cough	78 (17)
Weakness	47 (10)
Frequent hospital admission	32 (7)
Low CD4 count	18 (4)
New WHO III or IV disease	165 (36)
Previous elevated VL	794 (57)
VL after switch to 2nd line	50 (4)
Other	83 (6)
VL result	
Suppressed (<1000 copies/mL)	585 (39)
Elevated VL (≥1000 copies/mL)	926 (61)

* Patients who receive primary care at LH and had documented results during the testing period.

### Time to Clinical Action

Of the 926 patients with an elevated VL, 58% (475/816) patients were women with a mean age of 32 years. Seventy-eight percent (719/926) patients had a documented clinical action captured in the register: 77% (557/719) patients were switched to second line, whereas 15% (104/719) of patients were referred to intensified adherence counseling (Table 2). Seventy-eight percent (567/687) of the clinical actions occurred on the same day of testing, whereas 10% (67/687) occurred on the next day. Next day action was primarily due to patients arriving for care and having samples collected in the afternoon, in which case tests were run overnight and the results collected and acted on the next day. Eighty-two percent (450/537) of patients were switched to second line on the same day of testing. There was no difference in time to action for drug switch compared with referral for adherence counseling, with 76% and 84% action taken within the same day, respectively.

**TABLE 2.** Clinical Management and Time to Action for Patients With an Elevated VL (VL ≥ 1000 Copies/mL)

	N (%)
Elevated VL (≥1000 copies/mL)	926
Demographics	
Sex, female	475 (58)
Age [mean (IQR)]	32 (22–42)
“Clinical action” taken on a high VL result, n (%)	719 (78)
Referral for adherence counselling	104 (15)
Switch to 2nd line	557 (77)
No action*	19 (3)
Other	39 (5)
Time from sample collection to clinical action*	719 − 32 missing = 687
Same day	567 (82)
Next day	67 (10)
3 d–1 wk	29 (4)
8 d–1 mos	11 (2)
>2 mos	13 (2)
Time from sample collection to switch to 2nd line	557 − 20 missing = 537
Same day	450 (84)
Next day	51 (9)
3 d—1 wk	19 (4)
8 d–1 mos	8 (1)
>2 mos	9 (2)

* A “no action” response was provided, which is in contrast to those where we have no documentation for (926 − 719 = 207).

### Cost of Near-POC VL Testing

**FIGURE 1.**
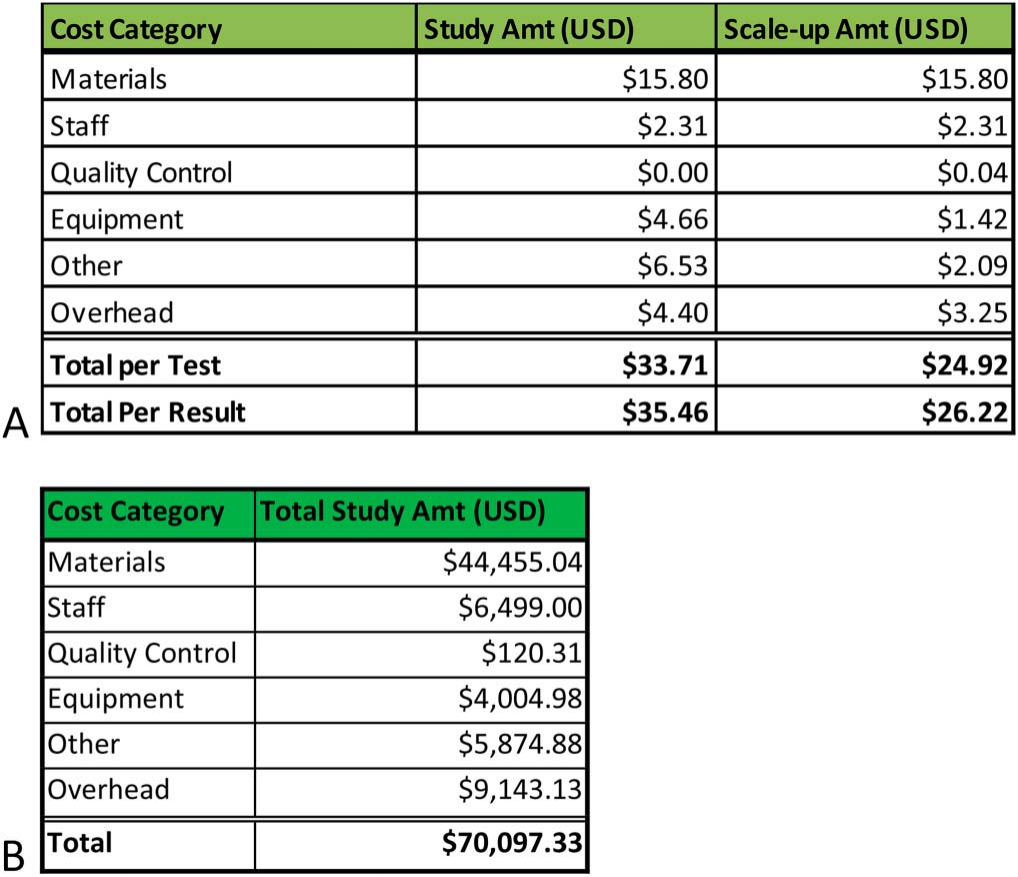
A, Cost per test and per result broken down by the main categories of cost components for the study and in a scale-up scenario. B, Cost per category for running the program for the year of 2017, including startup costs and the 2813 POC tests run during this intervention.

The all-in cost per POC VL test in the study was $33.71, which increased to $35.46 for a valid test result accounting for the need to repeat any test that resulted in an error. Using the study data, the cost for national scale-up has been estimated, assuming an optimized testing environment with 75% device utilization; the cost per test falls to $24.92 and $26.22 per valid result, accounting for errors (Fig. 1).

Materials, particularly the cost of the GeneXpert cartridge, accounted for the largest portion, 47% ($15.80) of the cost per test. The remaining 53% of the test cost comprised staff, equipment, other, and overhead. The other category includes freight, clearing and duty, initial training of device users, refresher trainings, and waste management, the largest of which is clearing and duty at $6.35 per test. The scale-up costs assumes 75% device utilization, whereas including estimates for costs not incurred in the study, such as external quality assurance and connectivity to enable remote monitoring of device performance. A sensitivity analysis shows that in the best-case scenario, the all-in cost per patient result could be as low as $21.03 and in a worst-case scenario as high as $60.15 per result. The primary driver of lower costs would be full utilization of the devices, running more tests than were observed during the study period. The main driver of increased cost in the worst-case scenario are primarily due to higher human resources cost from health workers not multitasking while assays are in the analyzer for 104 minutes and lower device utilization.

## DISCUSSION

The implementation of near-POC testing for targeted VL monitoring proved feasible at 2 high-volume urban clinics in Malawi. Near-POC VL testing enabled prompt and effective clinical management for nonpregnant adult and adolescent patients on ART who were suspected of treatment failure. Among patients deemed eligible for targeted testing, clinicians documented that 57% had a previously elevated VL and 33% had clinical signs or symptoms consistent with advanced disease or a serious coinfection such as tuberculosis. Sixty-one percent of patients tested using GeneXpert were found to have an elevated VL that required clinical attention, adherence counseling, or switch to second line. Across both clinics, 78% of patients with an elevated VL result had a documented clinical action, 82% of which happened on the same day as testing and 92% within 2 days of testing.

These findings illustrate several potential advantages of near-POC VL testing, particularly prompt data-driven clinical action. First, had patients perceived to be failing been presumptively switched to second line without the use of VL testing up to 39% (585/1511) may have been unnecessarily switched, indicating that the access to on-site VL testing enables more appropriate clinical action. Second, although only 2% of ART patients were receiving a second-line (protease inhibitor based) regimen at the end of 2017 throughout Malawi,^30^ in the same year 6% of VL tests performed nationally were >1000 copies/mL.^31^ Although not a one-to-one comparison and not all patients with an elevated VL should be switched to second line, the proportion of patients on second line is much lower than the proportion who have an elevated VL. In our study using GeneXpert for targeted POC VL testing, most patients with an elevated VL received a clinical action; 60% patients were switched to second line and 11% patients initiated adherence counseling, indicating the impact that rapid, immediate testing can have on effective clinical decision making in a nonresearch setting. Third, the same day clinical action not only benefits patients' health but also may reduce their out-of-pocket cost of returning to collect their test results at subsequent visits.

Assuming 240 working days per year, the average daily number of VL tests run was 5.7 at LH-KCH and 6.0 at LH-MPC; the average daily utilization during the study period was 6 tests, compared with a total theoretical throughput of 28 per day based on the 11-hour workday 5 days per week observed at the study sites. Comparing the cohort sizes, at LH-KCH, 1 near-POC VL was performed per 7.9 ART patients per year, and at LH-MPC, the ratio was 1 near-POC VL per 15.0 ART patients per year. With targeted testing in our cohort in high-volume centers of excellence, we reached only 32% utilization of the testing capacity of the GeneXpert devices, leaving plenty of capacity for other types of urgent near-POC testing that could be performed.

The cost per test was $33.71 and cost per result, accounting for test errors, increased to $35.46. With 68% remaining capacity, economies of scale dictate the cost per test can continue to be reduced.

In this study, the POC VL was mainly targeted to patients who had either a previous elevated VL, were suspected of advanced disease, or required a follow-up VL after switching to second line. In addition to these priority populations, the POC VL can be further expanded to other targeted populations, for example, pregnant and breastfeeding women^32^ or children and adolescents. Finally, near-POC VL testing would be useful in a remote or difficult-to-access location, which may not be part of routine sample transportation routes.

Several test types can be performed on the GeneXpert, such as EID, tuberculosis (TB), and human papillomavirus (HPV). Multidisease testing on GeneXpert across HIV VL, EID, and TB has been shown feasible in Zimbabwe.^33^ Indeed, a large fleet of GeneXpert devices already exists in many resource-limited settings mostly for TB testing,^34,35^ which could be leveraged for targeted VL testing to benefit more patients living with HIV on ART, without requiring large capital investments in new devices.

There are several limitations of our study, including that this is an observational study with no concurrent comparator in the project facilities. The implementation of the study in routine settings was intentional for purposes of assessing feasibility and process outcomes; however, record keeping was not optimal; we were only able to collect data for 54% of all POC VL test of interest. There is no reason to assume that the patients without test process documentation were systematically different from those with documentation. In addition, during the time of implementation, patient files without recent VL tests were actively flagged for testing, which likely led to overall increases in testing coverage during the study period, but may have not influenced testing among the targeted population substantially.

The most encouraging findings of this intervention were the reduced time between VL sample collection and management actions for patients eligible for a targeted near-POC VL, with most clinical decisions occurring on the same or next day as sample collection as well as the very high proportion of at-risk clients who had a documented clinical action after their VL testing. Even at very high-volume centers of excellence, the less than 10 at-risk patients were identified each day—well within the testing capacity of a 4 module GeneXpert.

This study shows the potential benefit of providing targeted near-POC VL technology for people in need of urgent clinical management, whereas most patients who require more routine clinical decisions receive centralized testing. Because of economies of scale, centralized VL testing is lower cost and may provide more consistent quality results and fewer operational challenges. It offers a valuable option for VL testing for most people on ART in lower resource settings who are stable and virally suppressed. Patients at risk of advanced disease, however, can feasibly be provided with and benefit from access to near-POC VL testing. The higher costs and facility resource required by near-POC testing are minimized by only using the intervention for the patients who will most benefit from prompt clinical action, including appropriate switch to a virally suppressing regimen. These costs can be further amortized by expanding the eligibility criteria for near-POC VL or using GeneXpert machines for additional purposes such as TB diagnoses. Therefore, targeted near-POC VL testing for the facilitation of high-quality patient care was both affordable and feasible. Further evaluation of how best to optimize use of near-POC VL with centralized VL testing, especially in the context of a multidisease testing program, is warranted.

## References

[1] WHO. Consolidated Guidelines on the Use of Antiretroviral Therapy for Treating and Preventing HIV Infection: Recomendations for a Public Health Approach. 2nd ed Geneva, Switerland: World Health Organization; 2016. 27466667

[2] UNAIDS. 90–90–90—an Ambitious Treatment Target to Help End the AIDS Epidemic. Geneva, Switerland: UNAIDS; 2014.

[3] UNAIDS. 2017 Global HIV Statistics. Geneva, Switerland: UNAIDS; 2018.

[4] UNAIDS. Joint United Nations Programme on HIV/AIDS (UNAIDS) DATA 2017. Geneva, Switerland: UNAIDS; 2017.

[5] WHO. HIV Diagnostic Tests in Low- and Middle-Income Countries: Forecasts of Global Demand for 2014–2018. 2015 Available at: http://www.who.int/hiv/pub/amds/diagnostic-forecast2014-2018/en/. Accessed December 7, 2019.

[6] Peter T Ellenberger D Kim AA Early antiretroviral therapy initiation: access and equity of viral load testing for HIV treatment monitoring. Lancet Infect Dis. 2017;17:e26–e29. 27773596 10.1016/S1473-3099(16)30212-2 PMC5745573

[7] Lecher S Williams J Fonjungo PN Progress with scale-up of HIV viral load monitoring—seven sub-Saharan African countries, 2015–2016. MMWR Morb Mortal Wkly Rep. 2016;65:1332–1335. 27906910 10.15585/mmwr.mm6547a2

[8] Ministry of Health—Malawi. Integrated HIV Program Report October—December 2016. Lilongwe, Malawi: Ministry of Health; 2016.

[9] Keiser O Chi BH Gsponer T Outcomes of antiretroviral treatment in programmes with and without routine viral load monitoring in Southern Africa. AIDS. 2011;25:1761–1769. 21681057 10.1097/QAD.0b013e328349822f PMC3605707

[10] Rowley CF Developments in CD4 and viral load monitoring in resource-limited settings. Clin Infect Dis. 2014;58:407–412. 24218101 10.1093/cid/cit733 PMC3890339

[11] Phillips A Shroufi A Vojnov L Sustainable HIV treatment in Africa through viral-load-informed differentiated care. Nature. 2015;528:S68–S76. 26633768 10.1038/nature16046 PMC4932825

[12] Ministry of Health Malawi. Clinical Management of HIV in Children and Adults. Lilongwe, Malawi: Ministry of Health; 2016.

[13] Prust ML Banda CK Nyirenda R Multi-month prescriptions, fast-track refills, and community ART groups: results from a process evaluation in Malawi on using differentiated models of care to achieve national HIV treatment goals. J Int AIDS Soc. 2017;20(suppl 4):21650. 28770594 10.7448/IAS.20.5.21650 PMC5577715

[14] Awungafac G Amin ET Fualefac A Viral load testing and the use of test results for clinical decision making for HIV treatment in Cameroon: an insight into the clinic-laboratory interface. PLoS One. 2018;13:e0198686. 29889862 10.1371/journal.pone.0198686 PMC5995384

[15] Etoori D Ciglenecki I Ndlangamandla M Successes and challenges in optimizing the viral load cascade to improve antiretroviral therapy adherence and rationalize second-line switches in Swaziland. J Int AIDS Soc. 2018;21:e25194. 30350392 10.1002/jia2.25194 PMC6198167

[16] Pai NP Vadnais C Denkinger C Point-of-care testing for infectious diseases: diversity, complexity, and barriers in low- and middle-income countries. PLoS Med. 2012;9:e1001306. 22973183 10.1371/journal.pmed.1001306 PMC3433407

[17] Peter T Zeh C Katz Z Scaling up HIV viral load—lessons from the large-scale implementation of HIV early infant diagnosis and CD4 testing. J Int AIDS Soc. 2017;20(suppl 7):e25008. 10.1002/jia2.25008 PMC5978645 29130601

[18] UNITAID. HIV/AIDS Diagnostics Technology Landscape. 5th ed Geneva, Switerland: Organization WH; 2015.

[19] Vojnov L Markby J Boeke C POC CD4 testing improves linkage to HIV care and timeliness of ART initiation in a public health approach: a systematic review and meta-analysis. PLoS One. 2016;11:e0155256. 27175484 10.1371/journal.pone.0155256 PMC4866695

[20] Mwenda R Fong Y Magombo T Significant patient impact observed upon implementation of point-of-care early infant diagnosis technologies in an observational study in Malawi. Clin Infect Dis. 2018;67:701–707. 29490026 10.1093/cid/ciy169 PMC6093992

[21] Jani IV Meggi B Loquiha O Effect of point-of-care early infant diagnosis on antiretroviral therapy initiation and retention of patients. AIDS. 2018;32:1453–1463. 29746301 10.1097/QAD.0000000000001846

[22] Bianchi F Cohn J Sacks E Evaluation of a routine point-of-care intervention for early infant diagnosis of HIV: an observational study in eight African countries. Lancet HIV. 2019;6:e373–e381. 30987937 10.1016/S2352-3018(19)30033-5

[23] Drain PD Violette L Quame-Amaglo J Point-of-care Viral Load Testing Improves HIV Viral Suppression and Retention in Care. Conference on Retroviruses and Opportunistic Infections; March 4–7, 2019; Seattle, Washington; 2019.

[24] Nicholas S Poulet E Wolters L Point‐of‐care viral load monitoring: outcomes from a decentralized HIV programme in Malawi. J Int AIDS Soc. 2019;22:e25387. 31441242 10.1002/jia2.25387 PMC6706700

[25] Ministry of Health—Malawi. Malawi Population-Based HIV Impact Assessment (MPHIA) 2015–2016: First Report. Lilongwe, Malawi: Ministry of Health; 2017.

[26] Mungwira RG Divala TH Nyirenda OM A targeted approach for routine viral load monitoring in Malawian adults on antiretroviral therapy. Trop Med Int Health. 2018;23:526–532. 29505108 10.1111/tmi.13047 PMC5932246

[27] WHO. WHO Prequalification of in Vitro Diagnostics-Xpert HIV-1 Viral Load with GeneXpert. Geneva, Switzerland: World Health Organization, 2017.

[28] Garrett NJ Drain PK Werner L Diagnostic accuracy of the point-of-care Xpert HIV-1 viral load assay in a South African HIV clinic. J Acquir Immune Defic Syndr. 2016;72:e45–e48. 26959192 10.1097/QAI.0000000000000978 PMC4866899

[29] Nash M Huddart S Badar S Performance of the Xpert HIV-1 viral load assay: a systematic review and meta-analysis. J Clin Microbiol. 2018;56:e01673–e01717. 29386266 10.1128/JCM.01673-17 PMC5869835

[30] Ministry, of Health M. Integrated HIV Program Report January–March 2018. Lilongwe, Malawi: Ministry of Health; 2018.

[31] Ministry of Health M. Viral Load Monitoring Cohort Report 2017. Lilongwe, Malawi: Ministry of Health; 2018.

[32] Myer L Essajee S Broyles LN Pregnant and breastfeeding women: a priority population for HIV viral load monitoring. PLoS Med. 2017;14:e1002375. 28809929 10.1371/journal.pmed.1002375 PMC5557351

[33] Ndlovu Z Fajardo E Mbofana E Multidisease testing for HIV and TB using the GeneXpert platform: a feasibility study in rural Zimbabwe. PLoS One. 2018;13:e0193577. 29499042 10.1371/journal.pone.0193577 PMC5834185

[34] Cazabon D Pande T Kik S Market penetration of Xpert MTB/RIF in high tuberculosis burden countries: a trend analysis from 2014–2016. Gates Open Res. 2018;2:35. 30234198 10.12688/gatesopenres.12842.1 PMC6139378

[35] Albert H Nathavitharana RR Isaacs C Development, roll-out and impact of Xpert MTB/RIF for tuberculosis: what lessons have we learnt and how can we do better? Eur Respir J. 2016;48:516–525. 27418550 10.1183/13993003.00543-2016 PMC4967565

